# Evaluation of Plasma Asprosin Concentration in Patients with Coronary Artery Disease

**DOI:** 10.21470/1678-9741-2021-0003

**Published:** 2022

**Authors:** Cengiz Güven, Hüseyin Kafadar

**Affiliations:** 1 Department of Cardiovascular Surgery, Faculty of Medicine, Adıyaman University, Adıyaman, Turkey.; 2 Department of Forensic Medicine, Faculty of Medicine, Adıyaman University, Adıyaman, Turkey.

**Keywords:** Asprosin, Coronary Artery Disease, Coronary Angiography, Severity of Illness Index, Risk Factors

## Abstract

**Introduction:**

The goal of this study is to investigate the association between diagnosis and severity of coronary artery disease (CAD) and Asprosin level.

**Methods:**

Patients diagnosed with CAD who underwent conventional coronary angiography for the first time were included in the present study. The patients were divided into four groups, each consisting of 20 individuals, as medical group, single coronary lesion group, double coronary lesion group, and multiple coronary lesions group. Serum Asprosin values and Gensini scores of the groups were compared in terms of compliance.

**Results:**

In this study, Asprosin values were found to be significantly higher in the multiple coronary lesions group than in the medical, single coronary, and double coronary lesion groups (*P*<0.05). In the double coronary lesion group, Asprosin values were significantly higher (*P*<0.05) than the in the medical and single coronary lesion groups. It was also found that Asprosin values did not differ at significant levels in the medical and single coronary lesion groups (*P*>0.05). It was determined that the increases in Asprosin values and Gensini scores were compatible with each other.

**Conclusion:**

The present study showed that the increases in serum Asprosin levels, along with the increases in the number of coronary arteries with critical stenosis, might be a marker in diagnosing and determining the severity of CAD.

**Table:** 

Abbreviations, acronyms & symbols	
BMI	= Body mass index
CAD	= Coronary artery disease
CCA	= Conventional coronary angiography
CI	= Confidence interval
SD	= Standard deviation

## INTRODUCTION

Coronary artery disease (CAD) is a clinical condition that is characterized by the stenosis and obstruction of the coronary arteries caused by atherosclerosis. Acute myocardial infarction and mortality might occur with the progression of the disease^[1]^.

Approximately 18 million deaths occur on an annual scale because of cardiovascular diseases, especially CAD. CAD ranks second in mortality reasons after traumatic deaths on a global scale. Deaths because of CAD are increasing rapidly in developing countries^[2^,^3]^. Early diagnosis and determining the prevalence of atherosclerosis play important roles in reducing cardiovascular diseases and related deaths^[4]^.

Interventional and non-interventional methods, such as conventional coronary angiography (CCA), computed tomography, exercise echocardiography, myocardial perfusion scintigraphy, cardiac magnetic resonance, and positron emission tomography can be used in the diagnosis of CAD^[5]^. When it is considered that only one-third of patients are diagnosed with CAD after undergoing CCA, which is the gold standard exam in our present day, it is important to use biochemical markers to determine the prevalence of the disease^[6]^.

A hormone with a protein structure has been discovered in recent years regulating the release of hepatic glucose and is the C-terminal division product of the profibrillin-1 protein. This hormone is secreted from white adipose tissue and increases the release of glucose and insulin during hunger. It is also known to stimulate the hypothalamic feeding center, leading to the stimulation of appetite and fat storage^[7]^.

In some previous studies conducted on this hormone, it was shown that it contributes to wound healing, has protective effects on the myocardium, and improves left ventricular functions significantly^[8^,^9]^.

Also, in an experimental study conducted on cardiovascular diseases, it was reported that Asprosin can improve cardiac microvascular endothelial damage in diabetic mice^[10]^. And in another study, it was shown that heart mesenchymal stromal cells were more resistant to apoptosis caused by both *in vitro* oxidative stress and *in vivo* myocardial ischemia in mice previously treated with Asprosin^[11]^.

Deaths because of CAD rank the first among the leading natural causes of death. We believe that this study will contribute to the literature because it shows that Asprosin may be a marker for diagnosing CAD and determining its severity with increased serum Asprosin levels as the number of coronary arteries with critical stenosis increases.

## METHODS

The study commenced with the permission (2019/2-5) on 20/03/2019 of the Adıyaman University, Faculty of Medicine, Non-Interventional Clinical Research Ethics Committee. Non-diabetic patients (hemoglobin A1c < 5.7%) who underwent elective coronary angiography with CAD pre-diagnosis at Adiyaman University, Faculty of Medicine, Cardiology Clinic between March 30, 2019, and August 30, 2019, and who provided voluntary informed consent forms were included in the present study. Body mass index > 30 (obese and morbidly obese) and diabetogenic diseases, such as primary hyperlipidemia diseases, endocrinopathy, and autoimmune metabolic diseases, were the exclusion criteria.

Patients were divided into four groups each consisting of 20 individuals: patients who had normal coronary angiography findings or who did not have critical lesions, which were included in the medical group; patients with a single critical coronary artery lesion (stenosis level > 75%) (single coronary lesion group); and patients with double (double coronary lesion group) and multiple (multiple coronary lesion group) critical coronary artery lesions.

The Gensini scoring system was used to measure the prevalence and severity of the disease in the coronary arteries. In this system, score ≤ 20 shows normal coronary artery, and patients with score > 20 are diagnosed with CAD. CAD intensity is given one point for 1-25% stenosis, two for 26-50% stenosis, four for 51-75% stenosis, eight for 76-90% stenosis, 16 for 91-99% stenosis and in the presence of antegrade flow, and 32 points for 100% stenosis. The score is limited to 32 in consecutive lesions for the same artery. Then, it is calculated by multiplying the standard multipliers determined by the lesion area^[12]^.

The blood samples used in the study were taken from the brachial vein right after elective coronary angiography. Serum samples were separated within half an hour, centrifuged at NüveNF 1200® brand centrifuge device at 1200 g × 3000 rpm for 10 minutes, and maintained at-40°C until the analyses day. After the samples were completed, Asprosin values were examined with enzyme-linked immunosorbent assay kit SEA332Hu (Cloud-Clone Corp., Katy, Texas, United States of America) for human Asprosin.

Asprosin hormone levels might vary with factors such as hunger, satiety, and circadian rhythm. For this reason, there is no definitive reference range in the literature. Its levels increase with hunger and drop acutely with eating. In individuals with normal weight, serum Asprosin levels are approximately 8-16 nanograms/ml^[13]^. We took these values as the reference range in our study.

The statistical analyses were made by using IBM Corp. Released 2013, IBM SPSS Statistics for Windows, version 22.0, Armonk, NY: IBM Corp and Medcal 9.0. Mean values, standard deviations, median, lowest, highest, frequency, and ratio values were used in the descriptive statistics of the study data. The distribution of the variables was measured with the Kolmogorov-Smirnov test. Kruskal-Wallis and Mann-Whitney U tests were used in the analysis of the independent quantitative data. Chi-squared (x^2^) test was used in the analysis of the independent qualitative data. Bland-Altman graph was used for the compliance of the measurements of Asprosin and Gensini values.

## RESULTS

**Fig. 1:**
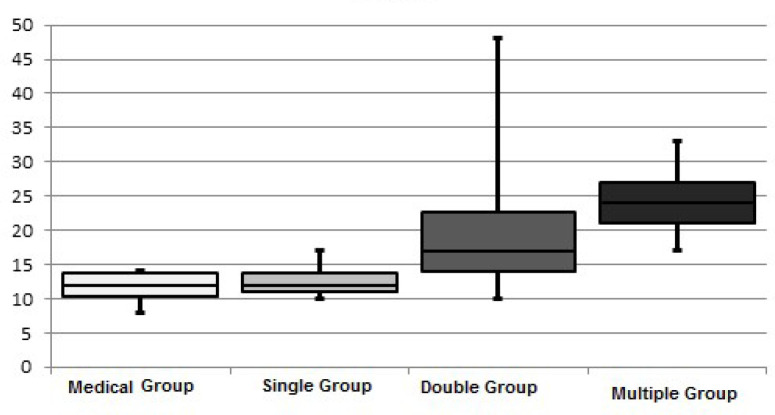
Asprosin levels between the groups.

A total of 80 patients were included in the study, which comprised of 24 women (30%) and 56 men (70%). The mean age of the patients was 61.7±11.5 years (Table 1). All patients had undergone elective coronary angiography with the pre-diagnosis of CAD. In the first group (medical group), nine of the 20 patients were female (45%), and 11 were male (55%). Asprosin values were found to be 11.7±1.8 in this group. It was found that three of the patients in the second group (single coronary lesion group) were female (15%), and 17 were male (85%); and serum Asprosin level was measured to be 12.2±2.1. A total of eight patients in the third group (double coronary lesion group) were female (40%), and 12 were male; and serum Asprosin was found to be 19.4±8.7. In the last group, there were four females (20%) and 16 males; and serum Asprosin was found to be 24.1±4.3 (Table 2). The increase in Asprosin values in the groups is summarized in Figure 1.

**Table 1:** Distribution of cases according to the patients’ gender.

		Min-Max	Median	Mean ± SD	n (%)
Age (years)		37-84	65	61.7±11.5	
Gender	Female				24 (30.0%)
	Male				56 (70.0%)
Asprosin		ago/48	14	16.8±7.2	

SD=standard deviation

**Table 2:** Demographic data and Asprosin distributions by groups.

			Medical	Single coronary lesion	Double coronary lesion	Multiple coronary lesions	*P*-value
Age (years)		Mean±SD	57.7±11.3	59.0±11.5	63.6±11.5	66.6±10.4	0.053^K^
		Median	57.0	58.5	63.0	69.0	
Gender	Female	n (%)	9 (45.0%)	3 (15.0%)	8 (40.0%)	4 (20.0%)	0.103^X2^
	Male	n (%)	11 (55.0%)	17 (85.0%)	12 (60.0%)	16 (80.0%)	
BMI		Mean±SD	25.8±2.4	26.5± 2.6	25.8±3.0	26.4±3.25	0.839^β^
		Median	25.6	26.8	26.2	25.3	
Asprosin		Mean±SD	11.7±1.8*^¥^	12.2±2.1*	19.4±8.7	24.1±4.3	0.000^K^
		Median	12.0	12.0	17.0	24.0	

K =Kruskal-Wallis (Mann-Whitney U test);^X2^=chi-squared test;

β =one-way analysis of variance;

¥ =difference with multiple coronary lesions group, P<0.05;

* =difference with double coronary lesion group, P<0.05

BMI=body mass index; SD=standard deviation

Statistically significant differences were detected between the ages of patients in the medical, single coronary, double coronary, and multiple coronary lesions groups (*P*>0.05). The gender distribution between the four groups did not show any significant differences (*P*>0.05) (Table 2).

In the multiple coronary lesions group, Asprosin values were significantly higher (*P*<0.05) than in the medical, single coronary, and double coronary lesion groups. Although Asprosin values were significantly higher in the double coronary lesion group than in the medical and single coronary lesion groups (*P*<0.05), they did not differ at significant levels in medical and single coronary lesion groups (*P*>0.05) (Table 2). Asprosin and Gensini score values of the groups are summarized in Table 3. No significant relations were detected between gender and Asprosin values (Table 4).

**Table 3:** Asprosin and Gensini score values/median (min-max).

Variables	Asprosin (n = 20)	Gensini (n = 20)
Medical	12 (8-14)	11 (2.5-14)
Single coronary lesion group	12 (10-17)	32 (22-58)
Double coronary lesion group	17 (10-48)	60 (48-98)
Multiple coronary lesions group	24 (17-33)	97 (68-112)

**Table 4:** Asprosin values by gender.

Variables	Gender	n (%)	Asprosin Mean ± SD	*P*-value^X2^
Medical group	Female	9 (45.0%)	11.72±1.96	0.717
	Male	11 (55.0%)	12.00±1.70	
Single coronary lesion group	Female	3 (15.0%)	11.67±1.70	0.789
	Male	17 (85.0%)	12.29±2.02	
Double coronary lesion group	Female	8 (40.0%)	22.12±10.30	0.321
	Male	12 (60.0%)	17.50±6.51	
Multiple coronary lesions group	Female	4 (20.0%)	22.75±4.91	0.154
	Male	16 (80.0%)	24.44±3.89	

^X2^=chi-squared test, SD=standard deviation

**Fig. 2:**
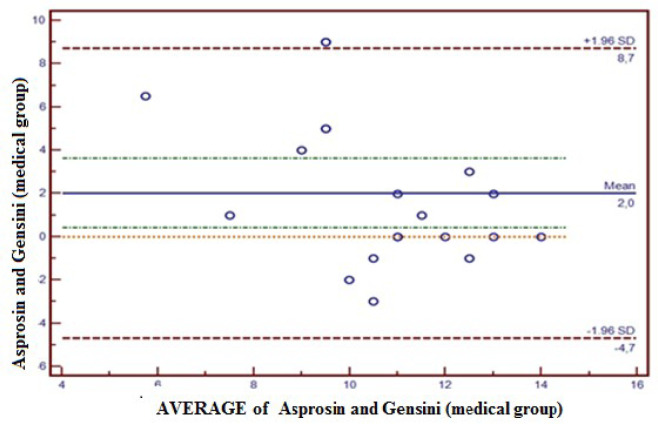
Concordance between Asprosin value and Gensini score in the medical group. The concordance between Asprosin and Gensini by Bland-Altman method in the medical group was-4.7 to 8.7 at a confidence level of 95%. Five percent (5%) of total observations was observed out of confidence level of 95%. SD=standard deviation

**Fig. 3:**
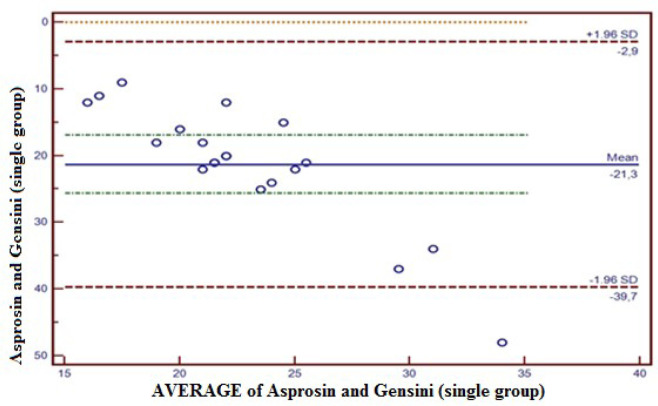
Concordance between Asprosin value and Gensini score in the single coronary lesion group. The concordance between Asprosin and Gensini by Bland-Altman method in the single coronary lesion group was-39.7 to 2.9 at a confidence level of 95%. Five percent (5%) of total observations was observed out of confidence level of 95%. SD=standard deviation

**Fig. 4:**
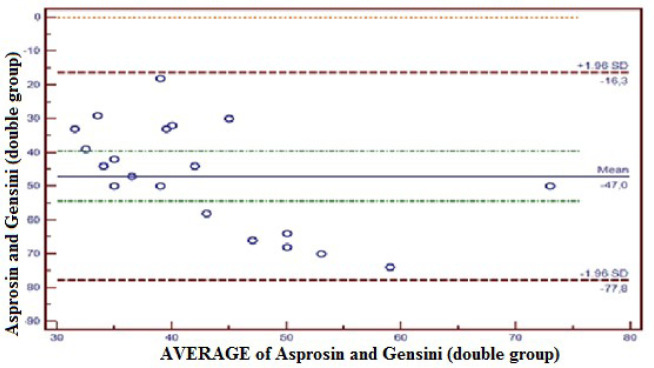
Concordance between Asprosin value and Gensini score in the double coronary lesion group. The concordance between Asprosin and Gensini by Bland-Altman method in the double coronary lesion group was-77.8 to 16.3 at a confidence level of 95%. Zero percent (0%) of total observations was observed out of confidence level of 95%. SD=standard deviation

**Fig. 5:**
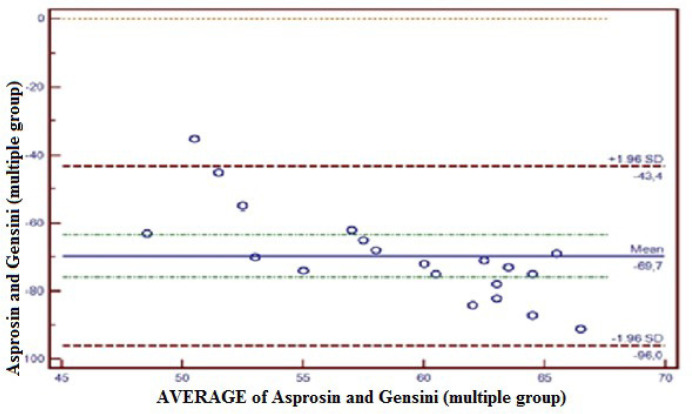
Concordance between Asprosin value and Gensini score in the multiple coronary lesion group. The concordance between Asprosin and Gensini by Bland-Altman method in the multiple coronary lesion group was-96.0 to 43.4 at a confidence level of 95%. Five percent (5%) of total observations was observed out of confidence level of 95%. SD=standard deviation

The Gensini scores were calculated for all patients, and the compliance between Gensini scores and Asprosin values was measured with the Bland-Altman plot method. In this respect, the compliance between Asprosin and Gensini in the medical group was within 95% confidence interval (CI) (-4.7 - 8.7); only 5% (1/20) of the total observations were outside the 95% CI (Figure 2). The compliance between Asprosin and Gensini in the single coronary lesion group was within 95% CI (-39.7 - 2.9); only 5% (1/20) of total observations were outside 95% CI (Figure 3). The compliance between Asprosin and Gensini in the double coronary lesion group was within 95% CI (-77.8 - 16.3); 0% (1/20) of total observations were outside 95% CI (Figure 4). The compliance between Asprosin and Gensini in the multiple coronary lesions group was within 95% CI (-96.0 - 43.4); only 5% (1/20) of total observations were outside 95% CI (Figure 5).

## DISCUSSION

The purpose of the present study was to uncover the relation between serum Asprosin levels and severity of CAD in the presence of CCA. Serum samples were collected from all patients participating in the study, and Asprosin levels were measured. The Gensini scores of all patients were calculated after CCA. The results were consistent with Gensini scores. In the medical group, the median min-max value was 11 (min-max: 2.5-14), and Asprosin level was 12 (min-max: 8-14). The Gensini score and serum Asprosin levels were measured as 32 (min-max: 22-58) and 12 (min-max: 10-17), respectively, in patients with single coronary artery lesion. In the double coronary artery lesion group, the Gensini score and serum Asprosin levels were 60 (min-max: 48-98) and 17 (min-max: 10-48), respectively. This increase was statistically significant (P<0.05). The Gensini score was 97 (min-max: 68-112) in patients with multiple coronary artery lesions. However, Asprosin levels were found to be 24 (min-max: 17-33), and this increase was statistically significant when compared to other groups. As it is understood from these values, the increase in the Gensini score and Asprosin levels was statistically related, which led us to the conclusion that there might be a relation between serum Asprosin levels and CAD severity.

CAD describes the stenosis or full obstruction status in coronary arteries caused by atherosclerosis, which, at a later stage, can result in myocardial infarction or death. Death due to CAD is the most common cause of non-traumatic mortality^[14^,^15]^. Early diagnosis and timely intervention might reduce mortality. Although there are many invasive and non-invasive methods employed for diagnosis, CCA is the most commonly used method^[4]^.

There is an increase in the tendency to carry out revascularization procedures in cardiac catheterization laboratories after diagnosis with noninvasive tests. Diagnostic biomarkers are used more often for this purpose^[16]^.

Although coronary angiography is the gold standard exam in the diagnosis of CAD, only one-third of patients who undergo total coronary angiography require intervention for critical stenosis; and this is the real reason for the search for other diagnostic methods. In this regard, low glomerular filtration rates and arrhythmia also restrict the use of CCA^[16]^.

Biochemical markers were also investigated and uncovered in CAD for diagnostic purposes as well as imaging tests. The most common molecules examined for this purpose are B-type natriuretic peptide, cardiac troponin I, cardiac troponin T, C-reactive protein, interleukin-6, gamma-glutamyl transferase, and glucagon-like peptide-1. These molecules were used for diagnostic purposes or to determine cardiac risk^[17^-^19]^.

Basically, Asprosin is secreted from white adipose tissue. This protein-based hormone is synthesized by the C-terminal part of the profibrillin-1 protein and secreted in case of hunger. The protein consists of 140 amino acids^[20]^.

Asprosin increases glucose and insulin release. Its levels decrease in case of satiety. Peripheral Asprosin crosses the blood-brain barrier, stimulates the hypothalamic appetite center, and ensures the maintenance of long-term fat. It is considered to be a glycogenic and orexigenic hormone with these effects^[7]^.

In the literature, an increase in Asprosin levels is found in studies conducted on people and mice that develop insulin resistance; Asprosin-specific antibodies resulted in decreased Asprosin concentrations, causing increased insulin sensitivity. It was emphasized that Asprosin can be used in the diagnosis of Type 2 diabetes with this effect^[21]^.

The SYNTAX score is one of the scoring systems that is employed for rating the anatomical severity of CAD. A study conducted by Yuan et al.^[20]^ showed that the changes in Asprosin levels in patients with the acute coronary syndrome were associated with SYNTAX score at significant levels. In the literature review, it was found that serum Asprosin levels were examined in some diseases, such as polycystic ovary syndrome and obesity besides CAD and diabetes, and it was emphasized that it might be a biochemical marker^[20]^. In another study, serum Asprosin levels were used to estimate the severity of unstable angina pectoris and acute coronary syndrome, and a significant increase was detected^[22]^.

The relation between Asprosin levels and gender is still a topic of discussion in the literature. Many studies conducted on adults showed that there were no differences^[23^-^25]^. Our study did not find any significant relations between gender and Asprosin values.

Gensini score alone provides an idea of CAD’s severity. However, coronary angiography is required for this^[10]^. This brings with it the negative effects of angiography. However, Asprosin value can be measured with a simple biochemical examination and provides an idea of the severity and prevalence of CAD.

### Limitations

The most important limitation of our study appears to be the relatively low patient population. Another limitation was the lack of examination of Asprosin values before coronary angiography. Whether the Asprosin value, which is affected by many factors, changes with a stressful process such as CCA can be evaluated by further studies.

## CONCLUSION

Asprosin and Gensini scores were calculated separately in the present study, and the compliance between them was compared with the Bland-Altman plot method. The result was found to be at 95% CI, which demonstrated the compliance of these two methods. As a conclusion, the data obtained in this study show that there is a direct proportion between serum Asprosin level and CAD severity. However, when it is considered that Asprosin hormone is a relatively new molecule, we believe that future studies with wider patient populations are needed.

**Table:** 

Authors' roles & responsibilities	
CG	Substantial contributions to the conception and design of the work; or the acquisition, analysis, or interpretation of data for the work; drafting the work or revising it critically for important intellectual content; final approval of the version to be published
HK	Substantial contributions to the conception and design of the work; or the acquisition, analysis, or interpretation of data for the work; drafting the work or revising it critically for important intellectual content; final approval of the version to be published
